# Transmembrane Domain Single-Nucleotide Polymorphisms Impair Expression and Transport Activity of ABC Transporter ABCG2

**DOI:** 10.1007/s11095-017-2127-1

**Published:** 2017-03-09

**Authors:** Noora Sjöstedt, Jeroen J. M. W. van den Heuvel, Jan B. Koenderink, Heidi Kidron

**Affiliations:** 1 Centre for Drug Research, Division of Pharmaceutical Biosciences, Faculty of Pharmacy, University of Helsinki, Viikinkaari 5E, 00014 Helsinki, Finland; 2 Department of Pharmacology and Toxicology, Radboud Institute for Molecular Life Sciences, Radboud University Medical Center, Nijmegen, The Netherlands

**Keywords:** BCRP, genetic variant, interindividual variability, pharmacogenetics, SNP

## Abstract

**Purpose:**

To study the function and expression of nine naturally occurring single-nucleotide polymorphisms (G406R, F431L, S441N, P480L, F489L, M515R, L525R, A528T and T542A) that are predicted to reside in the transmembrane regions of the ABC transporter ABCG2.

**Methods:**

The transport activity of the variants was tested in inside-out membrane vesicles from Sf9 insect and human derived HEK293 cells overexpressing ABCG2. Lucifer Yellow and estrone sulfate were used as probe substrates of activity. The expression levels and cellular localization of the variants was compared to the wild-type ABCG2 by western blotting and immunofluorescence microscopy.

**Results:**

All studied variants of ABCG2 displayed markedly decreased transport in both Sf9-ABCG2 and HEK293-ABCG2 vesicles. Impaired transport could be explained for some variants by altered expression levels and cellular localization. Moreover, the destructive effect on transport activity of variants G406R, P480L, M515R and T542A is, to our knowledge, reported for the first time.

**Conclusions:**

These results indicate that the transmembrane region of ABCG2 is sensitive to amino acid substitution and that patients harboring these ABCG2 variant forms could suffer from unexpected pharmacokinetic events of ABCG2 substrate drugs or have an increased risk for diseases such as gout where ABCG2 is implicated.

**Electronic supplementary material:**

The online version of this article (doi:10.1007/s11095-017-2127-1) contains supplementary material, which is available to authorized users.

## Introduction

The transporter ABCG2, often referred to as the breast cancer resistance protein (BCRP), is a member of the ATP-binding cassette (ABC) transporter family. ABC transporters are membrane proteins that use ATP to exclude a diverse range of molecules including endogenous metabolites and xenobiotics from cells. The *ABCG2* gene, located in chromosome 4, encodes a 655 amino acid long peptide chain that forms an intracellular N-terminal ATP-binding domain followed by six transmembrane helices. The detailed organization in the cell membrane remains to be elucidated since there is no available crystal structure of ABCG2. The structure of ABCG2 is different from typical ABC-transporters that have 12 or more transmembrane helices and two ATP-binding domains. Due to its “half-transporter” structure, ABCG2 has to form dimers to exert its transport function, but it has also been suggested to function as tetramers or higher order oligomers (1,2).

Expression of ABCG2 in cancer cells increases resistance to several chemotherapeutic agents such as mitoxantrone, topotecan and methotrexate (3–5). Due to its localization in the intestine, liver and kidneys (4,6), ABCG2 increases elimination of substrate drugs and decreases absorption and bioavailability. ABCG2 also has a protective role in limiting distribution of xenobiotics in distinct organ compartments such as the brain and placenta (4,7). In addition to transporting drugs and toxins, ABCG2 disposes of endogenous metabolites, including estrogen metabolites (8). ABCG2 is one of the transporters involved in uric acid transport in the body and dysfunction of ABCG2 is shown to be related to gout risk (9,10). On the other hand, ABCG2 null alleles have been linked to a Junior (a-) blood group phenotype that results in adverse effects in blood transfusions (11). It is suggested that ABCG2 and its variant forms could also be involved in photosensitivity, since it is involved in cellular porphyrin homeostasis (12). In addition to this, many more xenobiotic substrates as well as a plethora of inhibitors have been identified in *in vitro* studies, which means that there is a great potential for unwanted ABCG2-mediated pharmacokinetic events. A decrease in ABCG2 transport activity arising from interindividual genetic variations or inhibition can result in unexpectedly high concentrations of substrate drugs due to increased absorption and decreased elimination.

Nonsynonymous single-nucleotide polymorphisms (SNPs) introduce an amino acid change in the peptide sequence, which can alter the expression, function or localization of the proteins they encode. Genotyping studies of *ABCG2* have revealed several hundred naturally occurring nonsynonymous SNPs (424 variants listed in the Ensembl GRCh37 database, release Oct2016). The majority of these variants occur at a frequency of < 1% in most populations. The most common variants are V12M and Q141K, which cause amino acid changes in the intracellular regions of ABCG2. They occur at a frequency of 19.2% and 31.9%, respectively, in the Japanese population (9), but are rarer in other populations, with the Q141K minor allele frequency being roughly 10% in Caucasians and 3% in blacks (10,13). In addition to being linked to gout (9,10), the Q141K variant alters pharmacokinetics of atorvastatin, rosuvastatin and sulfasalazine (13,14). The decreased transport of Q141K, observed to be around 50% of wild-type (WT) ABCG2 in *in vitro* studies, can be explained by reduced plasma membrane expression (9,15–17). The other common variant, V12M, does not appear to confer changes in expression level or function of ABCG2 (9,12,15–17).

An acquired transmembrane domain SNP, resulting in the change of R482 to threonine or glycine, was identified in several cancer cell lines *in vitro.* It has received considerable interest, because it was shown to be a gain-of-function mutation leading to increased mitoxantrone and doxorubicin resistance compared to the WT as well as the ability to transport daunorubicin, rhodamine 123 and LysoTracker green, unlike WT ABCG2 (18–20). Discovery of the R482 mutant lead to a number of mutational studies on other transmembrane residues and decreased transport function of mitoxantrone resistance has been linked for example to changes at positions 336, 557 and 630 (19). These results indicate that changes in the transmembrane helices of ABCG2, which line the substrate binding cavity, can have a significant effect on substrate recognition.

**Fig. 1:**
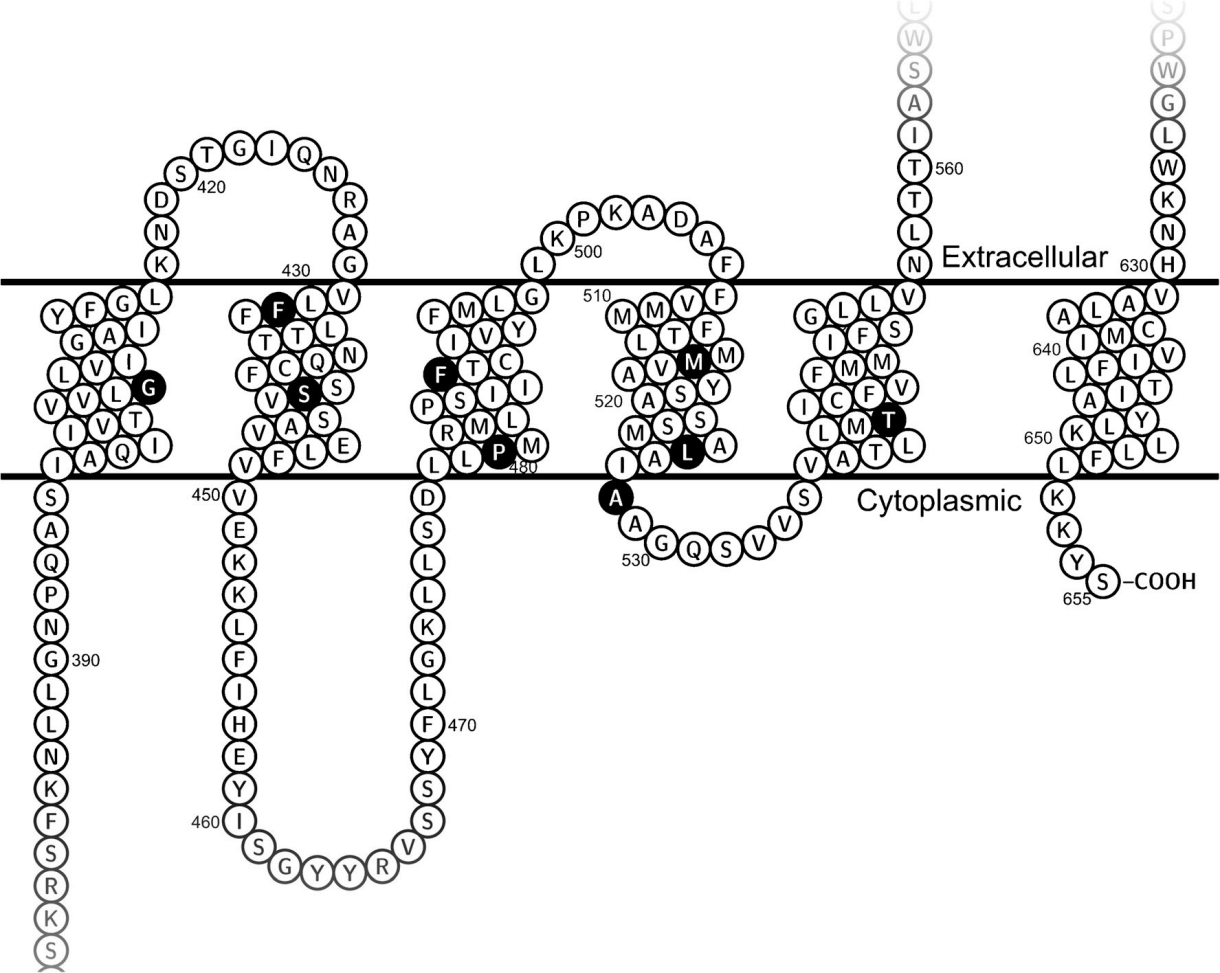
Predicted localization of the studied single-nucleotide polymorphisms in the transmembrane regions of ABCG2. The figure was generated using Protter (37), based on the Uniprot entry Q9UNQ0.

Here we present the function and expression of nine naturally occurring SNPs (G406R, F431L, S441N, P480L, F489L, M515R, L525R, A528T and T542A) that are predicted to reside in the transmembrane domains of ABCG2 and were therefore hypothesized to impact ABCG2 transport activity (Fig. 1, Table I). Indeed, we found that all nine studied variants had impaired substrate transport that could not be explained solely by expression levels. Patients harboring these variants may thus experience altered pharmacokinetics of ABCG2 substrate drugs and increased risk of diseases where ABCG2 is implicated.

**Table I:** Studied ABCG2 Variants and Predicted Consequences of the Amino Acid Changes

SNP	Amino acid change	Variant id	SIFT value (and consequence)^a^	Poly-Phen value (and consequence)^a^
1216 G > A	G406R	rs142628079	0.01 (Damaging)	0.936 (Possibly damaging)
1291 T > C	F431L	NA	0.12 (Tolerated)	0.584 (Possibly damaging)
1322 G > A	S441N	rs758900849	0.55 (Tolerated)	0.091 (Benign)
1439 C > T	P480L	rs202192122	0.02 (Damaging)	0.959 (Probably damaging)
1465 T > C	F489L	rs192169063	0.07 (Tolerated)	0.180 (Benign)
1544 T > G	M515R	rs199806536	0.02 (Damaging)	0.933 (Possibly damaging)
1574 T > G	L525R	rs58818712	0 (Damaging)	1.000 (Probably damaging)
1582 G > A	A528T	rs45605536	0.02 (Damaging)	0.198 (Benign)
1624 A > G	T542A	rs35965584	0.62 (Tolerated)	0.149 (Benign)

NA = not available

^a^ Consequences of the amino acid changes were predicted with the SIFT Human Protein online service (21) (http://sift.jcvi.org/www/SIFT_enst_submit.html) and the Poly-Phen 2 service (22) (http://genetics.bwh.harvard.edu/pph2/) using the human ABCG2 protein sequence (Uniprot entry Q9UNQ0) as the template.

## Materials & Methods

### Materials

[^3^H]-estrone sulfate ammonium salt (E_1_S; 54 Ci/mmol) was purchased from Perkin Elmer (Boston, MA, USA). Fetal bovine serum (FBS) and DMEM were from Gibco (Invitrogen, NY, USA) and HyClone SfX Insect cell medium from Thermo Fisher Scientific (Waltham, MA, USA). Lucifer yellow CH dipotassium salt (LY), unlabeled E_1_S and all other chemicals were from Sigma-Aldrich (St. Louis, MO, USA) unless stated otherwise. Mutagenesis primers (Supplementary 1) were produced by Oligomer (Helsinki, Finland).

### Preparation of Plasmids Carrying ABCG2 Variant Forms

The pFastBac1-BCRP vector and pENTR221-hBCRP entry vector for Gateway cloning were constructed as described previously (23,24). The SNPs were incorporated into the *ABCG2* gene in these vectors using the Q5 site-directed mutagenesis kit (New England Biolabs Inc., Ipswich, MA, USA) except for the 1582 G > A which was produced using the Q5 polymerase (New England Biolabs Inc.) and overlapping primers. DpnI (Thermo Fisher Scientific) was used to digest the template DNA. The presence of the SNPs was verified by sequencing the whole *ABCG2* gene using the sequencing service from GATC Biotech (Constance, Germany). The ABCG2 WT and variant genes were transferred from the pENTR221-hBCRP entry vector to a modified Bac-to-Bac vector using Gateway cloning (Invitrogen). Recombinant baculoviruses for both Sf9 and HEK293 expression were generated according to the Bac-to-Bac protocol (Invitrogen Life Technologies, Carslbad, CA, USA). A vector containing the gene for enhanced yellow fluorescent protein (eYFP) was used as a control in the HEK293 expression system and an empty bacmid served as a control in the Sf9 system.

### Cell Culture and Protein Expression

Sf9 cells were grown as a suspension culture in HyClone SfX Insect cell medium supplemented with 5% FBS at 27°C. For ABCG2 expression in Sf9 cells, the cells were harvested by centrifugation at 1000 g for 10 min (+4°C) 55 h after infection with recombinant baculovirus and washed once with phosphate buffered saline. HEK293 cells were cultured in high-glucose DMEM + GlutaMax-I supplemented with 10% fetal bovine serum at 37°C, 5% CO_2_. Confluent cells were split 1:3 into T175 flasks and transduced the next day with ABCG2 WT, ABCG2 variant or eYFP baculovirus preparations. Cells were incubated with the virus and 5 mM sodium butyrate for 72 h and then harvested by centrifuging at 3000 g for 15 min.

### Vesicle Preparation

Crude membrane vesicles were prepared from Sf9 cells with slight modifications to the method described by Chu *et al.* (25). After washing twice with buffer containing 50 mM Tris-HCl (pH 7.0) and 300 mM mannitol, cells were homogenized in membrane buffer (50 mM Tris-HCl (pH 7.0), 50 mM mannitol and 2 mM EDTA) using a low clearance Dounce homogenizer for 40 strokes. Lysed cells were centrifuged at 800 g for 10 min and membranes from the supernatant were pelleted by centrifugation at 100 000 g for 75 min at + 4°C. The pellet was suspended in membrane buffer. Similarly, HEK293-vesicles were produced by resuspending the cell pellet in Tris-sucrose (TS) buffer (10 mM TRIS-HEPES, 250 mM sucrose, pH 7.4) and homogenizing using the Dounce homogenizer as with Sf9 cells. The lysed cells were centrifuged at 4000 g for 20 min at + 4°C and the resulting supernatant was centrifuged for 90 min at 21,000 g, + 4°C. Pellets were suspended with TS buffer. For both HEK293 and Sf9 vesicle preparations, the resuspended crude membrane pellet was passed 20 times through a 27 gauge needle and the protein concentration of all vesicle preparations was measured using the Bio-Rad protein assay based on the Bradford method (Bio-Rad Laboratories Inc., Hercules, CA, USA). Membrane vesicles were frozen in aliquots in liquid nitrogen and stored at -80°C until use.

### Transport Assays

Vesicular transport assays were performed using the rapid filtration technique with Multiscreen_HTS_96-well, 1.0/0.65 μm pore, glass fiber filter plates (MSFBN6B50) Millipore (Molsheim, France) on a MultiScreenHTS vacuum manifold (Millipore). Sf9 transport assays were performed by pre-incubating vesicles (total protein amount 50 μg) with ABCG2 substrate Lucifer Yellow (LY) in buffer (40 mM MOPS-Tris (pH 7.0), 60 mM KCl and 6 mM MgCl_2_) for 5 min, then adding plain buffer (background control) or ATP to a final concentration of 4 mM. Reactions were terminated after 10 min using ice-cold washing mix (40 mM MOPS-Tris (pH 7.0) and 70 mM KCl) and immediately filtered. Filters were washed 5 times before drying. In the HEK293 transport assays, vesicles (total protein amount 15 μg) were incubated in TS buffer in the presence of 10 mM MgCl_2_, 4 mM ATP or AMP (negative control) and substrate at 37°C (LY) or 32°C (E_1_S). E_1_S was used as a mixture of ^3^H-labeled and unlabeled compound at a final concentration of 1 μM. A 50 μM solution of unlabeled E_1_S was used to pre-wet the filter plate to decrease unspecific binding of ^3^H-labeled E_1_S. Reactions were stopped after 2 min (E_1_S) or 10 min (LY) using ice-cold TS buffer, filtered and washed twice with 200 μl TS buffer.

For analysis, LY samples were eluted with 100 μl 0.1 M NaOH and neutralized with an equal amount of 0.1 M HCl. Fluorescence was detected using Varioskan Flash (Thermo Fisher Scientific, Vantaa, Finland) at 430 nm excitation and 538 nm emission. E_1_S samples were analyzed using scintillation counting with a Wallac 1450 Microbeta Trilux scintillation counter (Perkin Elmer, Boston, MA, USA) after addition of 50 μl of OptiPhase HiSafe 2 scintillation cocktail (Perkin Elmer) to each well.

### Western Blot

Vesicle samples (2 or 5 μg total protein per well) were mixed with 2 x Laemmli buffer (Bio-Rad Laboratories) and separated on a Mini-PROTEAN TGX Stain-Free 10% SDS-PAGE gel (Bio-Rad Laboratories). Samples were subsequently transferred to a 0.45 μm nitrocellulose membrane (Bio-Rad Laboratories). Membranes were blocked with 5% (w/v) skimmed milk solution. Anti-human ABCG2 mouse monoclonal antibody BXP-21 (ab3380, Abcam, Cambridge, United Kingdom) was used at a 1:5000 dilution in 5%(w/v) skimmed milk in TBS-Tween (0.1% Tween 20) to detect ABCG2. Goat Anti-Mouse IgG Antibody, (H + L) HRP conjugate (Millipore, Temecula, CA, USA) was used at 1:10 000 dilution in 2.5% (w/v) skimmed milk in TBS-Tween as the secondary antibody. Incubation time for both antibodies was 1 h. After washing with TBS-Tween and TBS, bands were visualized with the Amersham ECL Prime Western blotting detection reagent (GE Healthcare, Buckinghamshire, United Kingdom) and the ChemiDoc XRS+ system (Bio-Rad Laboratories). Image Lab software version 5.1 (Bio-Rad) was used to quantify the band intensities. The Bio-Rad Stain-free technology was used according to the manufacturer’s instructions to normalize the data for total protein content in lanes.

### Immunofluoresence Staining and Microscopy

HEK293 cells were seeded onto LabTek 8-well chamber slides coated with 0.1 mg/ml poly-D-lysine at 0.2 *10^5^ cells/well. After 24 h incubation, cells were transduced using the generated ABCG2 variant baculoviruses in the presence of 5 mM sodium butyrate. Cells were fixed the following day using 4% PFA. A 0.5% saponin-PBS solution was used to permeabilize cells and 10% goat serum in 0.1% saponin-PBS was used for blocking for 1 h. Cells were labeled with BXP-21 (1:2000) and subsequently with goat anti-Mouse IgG (H + L)-Alexa Fluor 488 secondary antibody (A-11001, ThermoFisher Scientific) at a 1:200 dilution. Normal mouse IgG (1:400, Santa Cruz Biotechnology, Santa Cruz, CA, USA) was used as a control. DAPI was used at 25 μg/ml to visualize the nuclei. Cells were imaged using a Leica DM6000B wide-field microscope (Leica Microsystems, Wetzlar, Germany) at 40 x magnification. Staining and microscopy was performed on two separate batches of HEK293 cells transduced with the recombinant baculoviruses.

### Data Analysis

ATP-dependent transport was calculated as the difference between the uptake in the presence and absence of ATP. Transport activity in the ABCG2 variant vesicles was normalized to the activity in wild-type ABCG2 vesicles. Statistical significance in activity differences was evaluated using one-way analysis of variance (ANOVA) with the Dunnett’s *post hoc* test for multiple comparisons. The same test was also used for the comparison of expression data from western blots. Non-linear regression was used to calculate V_max_ and K_m_ values for Lucifer Yellow kinetics in selected vesicle preparations. Statistical significance of the difference in K_m_ and V_max_ values was evaluated using the extra-sum-of-squares F-test. In all analyses, *p*-values below 0.05 were considered significant. All statistical and regression analyses were performed using GraphPad Prism 6.05 (GraphPad Software, San Diego, CA, USA).

## Results

Nine variant forms of ABCG2 were successfully generated and expressed in Sf9 and HEK293 cells. Transport activity of the variants was evaluated using the vesicular transport assay with probe substrates Lucifer Yellow (LY) and estrone sulfate (E_1_S). The expression levels of the variants in the membrane vesicle preparations were studied using western blotting and the intracellular localization was evaluated in HEK293 cells using immunofluorescence microscopy.

### Lucifer Yellow Transport in Sf9-ABCG2 Variant Vesicles

**Fig. 2:**
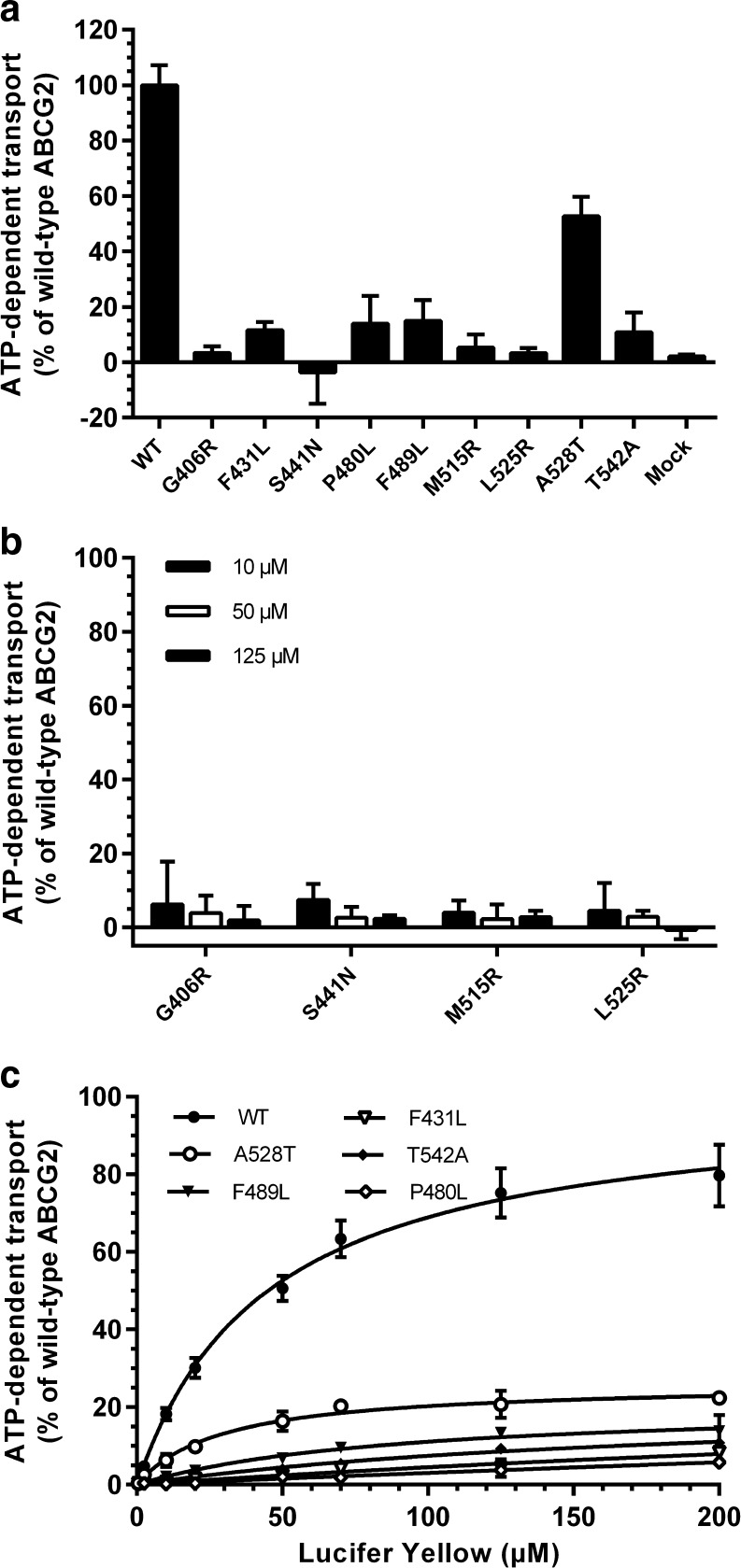
Transport of Lucifer Yellow (LY) into Sf9-ABCG2 variant vesicles (50 μg total protein/well). (**a**) Transport activity was assayed for all variants at 50 μM LY (**b**) Low transport activity was verified for the least active variants (uptake ratio < 1.5 at 50 μM LY) at 10, 50 and 125 μM LY. Data in (**a**) and (**b**) is presented as mean ATP-dependent transport (± SD) normalized to wild-type (WT) ABCG2 from two separate batches of vesicles with reactions performed in triplicates (**c**) Kinetics of LY transport. Incubation time with ATP was 10 min. Data is presented as ATP-dependent uptake normalized to the calculated V_max_ of WT ABCG2. Points represent mean ± SD of an experiment performed in triplicates. Transport activity was significantly different from WT ABCG2 in all preparations (*p* < 0.0001) according to one-way ANOVA analysis with the Dunnett’s *post hoc* test.

Eight out of the nine studied SNPs decreased LY transport at 50 μM by more than 75% compared to WT ABCG2 (Fig. 2a). The exception was the alanine to threonine change at position 528, which decreased transport to 53%. The same pattern of impaired transport for the variants was seen for E_1_S (data not shown)*.* The decrease in transport was verified with LY for the least active variants (G406R, S441N, M515R and L525R) at two additional concentrations (10 μM and 125 μM) (Fig. 2b). Variants F431L, P480L, F489L, A528T and T542A were subjected to more detailed studies of LY kinetics (Fig. 2c). Although a trend for Michaelis-Menten kinetics could be seen, reliable K_m_ values could only be calculated for the least impaired variant (A528T), where the K_m_ was 29.4 ± 4.67 μM. The change in K_m_ was not significantly different to the WT ABCG2 that has a K_m_ of 45.1 ± 4.85 μM, whereas the V_max_ of A528T was measured to be 26% of the WT, which is significantly lower (*p* < 0.0001).

### Lucifer Yellow and Estrone Sulfate Transport in HEK293-ABCG2 Variant Vesicles

**Fig. 3:**
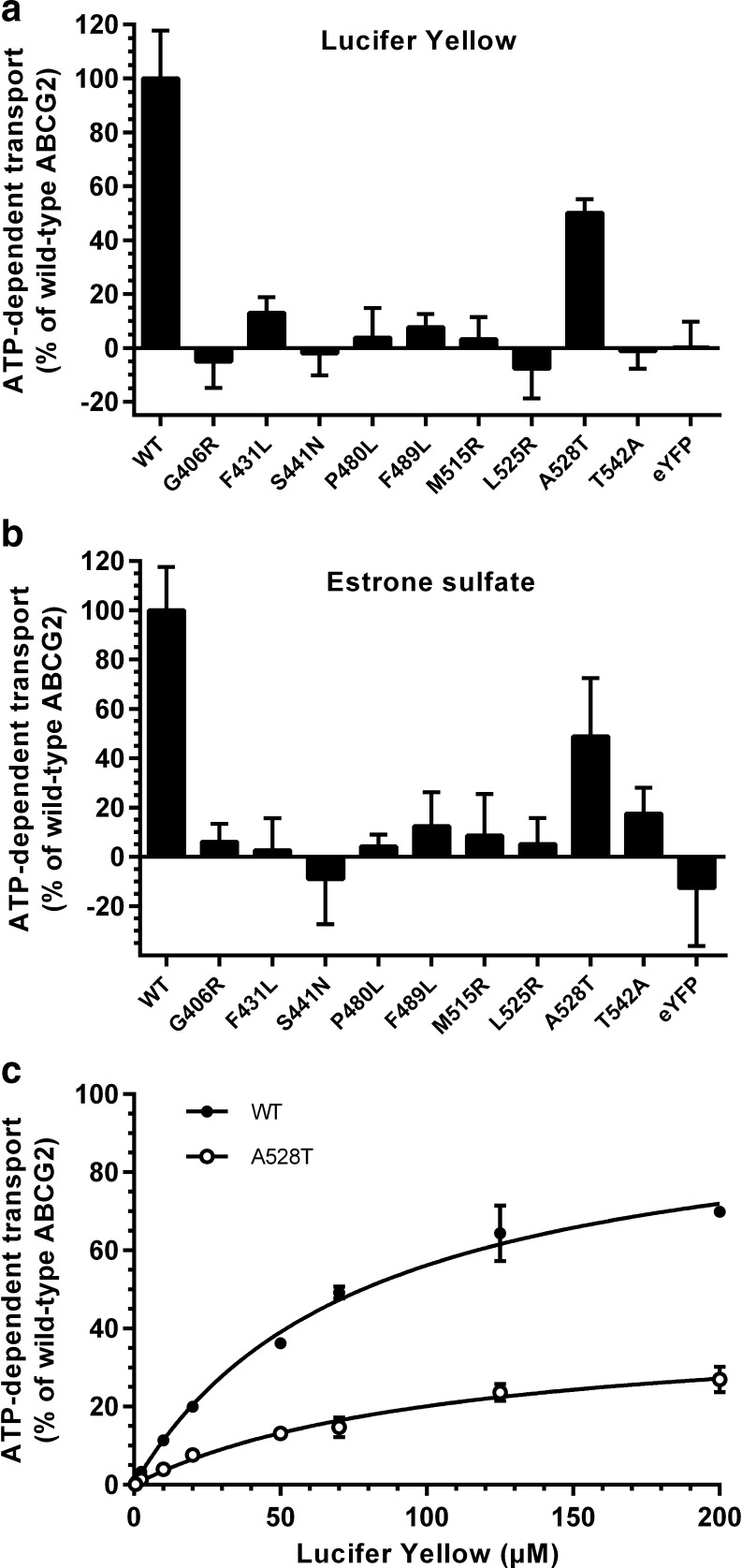
Transport of 50 μM Lucifer Yellow (**a**) and 1 μM estrone-sulfate (**b**) into HEK293-ABCG2 variant vesicles (15 μg total protein/well). Data in (**a**) and (**b**) is presented as ATP-dependent uptake normalized to uptake in wild-type (WT) ABCG2 vesicles. (**c**) Kinetics of Lucifer Yellow transport into HEK293-ABCG2 WT and A528T vesicles (15 μg total protein/well). Data is presented as ATP-dependent transport normalized to the calculated V_max_ of WT ABCG2. Points represent mean ± SD from experiments with two separate batches of vesicles with reactions performed in triplicates. In (**a**) and (**b**) transport activity was significantly different from WT ABCG2 in all preparations (*p* < 0.0001) according to one-way ANOVA analysis with Dunnett’s *post hoc* test.

The transport activity of LY in the HEK293-ABCG2 variant vesicles was in line with the results observed with Sf9 vesicles. With 50 μM LY, transport by A528T was decreased to 50% of the WT, but the transport activity of G406R, S441N, L525R and T542A was abolished and severely impaired for F431L, P480L and F489L (Fig. 3a). The variants showed similar impaired transport activity of the physiological substrate E_1_S (1 μM), although for the T542A variant we observed transport of E_1_S, while no LY transport was detected (Fig. 3b). The transport activity of A528T was similar with both substrates. The V_max_ of LY transport for the A528T variant was 42% of WT ABCG2 (*p* < 0.001) (Fig. 3c). The calculated K_m_ values were higher than in Sf9 vesicles, 78.1 ± 9.07 μM and 108 ± 22.0 μM for WT and A528T, respectively, but as with the Sf9 preparations, the difference in K_m_ between WT and A528T was not significant.

### Expression and Localization of ABCG2 Variants

**Fig. 4:**
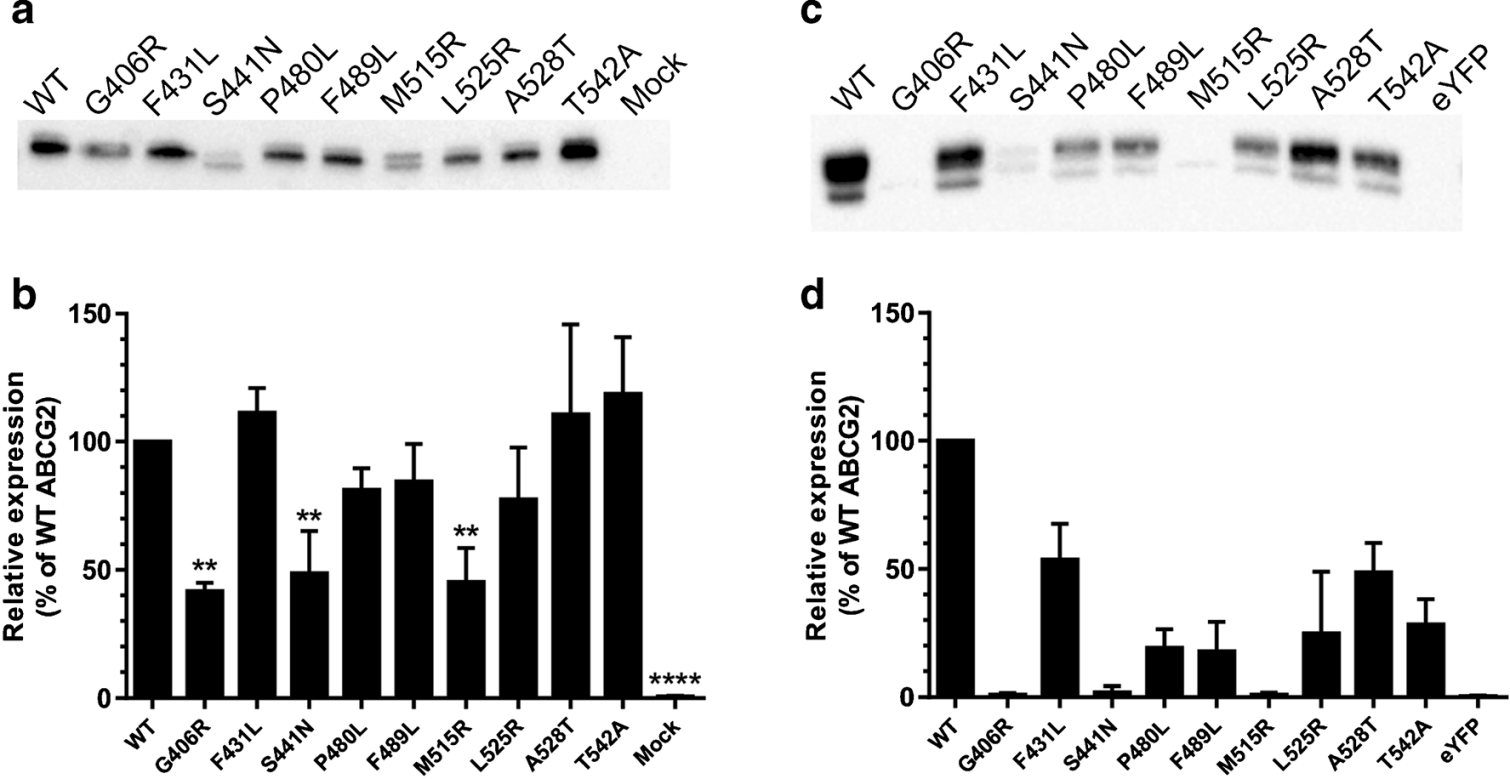
Expression of ABCG2 variants in vesicles produced from infected Sf9 or transduced HEK293 cells. (**a**) and (**c**) western blot analysis of ABCG2 expression levels in isolated crude membrane vesicle preparations from Sf9-ABCG2 (**a**) and HEK293-ABCG2 cells (**c**) and corresponding control vesicles (mock and eYFP). Representative blots are shown. (**b**) and (**d**) Expression levels of the variants based on band intensity quantification of western blots using the Image Lab software from Bio-Rad for (**b**) Sf9-ABCG2 vesicles and (**d**) HEK293-ABCG2 vesicles. Band intensity was normalized using the total protein signal from lanes and results are presented as the relative expression (%) compared to WT ABCG2. Bars represent the mean relative expression (± SD) from triplicate western blots. The statistical significance of differences compared to the WT was analyzed using one-way ANOVA with the Dunnett’s *post hoc* test. In (**b**) ** *p* < 0.001, **** *p* < 0.0001 and in (**d**) *p* < 0.0001 for all variants except F431L, where *p* < 0.001.

Western blotting was used to study the expression levels of ABCG2 variants in the membrane vesicles used for the activity studies. ABCG2 was detected using the BXP-21 antibody, which reacts with the intracellular domains of ABCG2 (amino acid residues 271-396) and therefore the presence of the studied amino acid changes are not expected to change the affinity of the antibody. ABCG2 protein could be detected in all of the Sf9 and HEK293 vesicle preparations of ABCG2 variants, but the levels in HEK293 vesicles were lower than in Sf9 (Fig. 4). In Sf9 vesicles, six of the variants were expressed at levels above 50% of WT, whereas 54% was the highest relative expression achieved with any of the variants in the HEK293 vesicles. The expression of G460R, S441N and M515R was significantly lower than the WT (*p* < 0.01) in the Sf9 vesicles and only negligible amounts (<2% of WT) of these three variants could be detected in the HEK293 vesicles. All of the variants expressed in HEK293 cells showed significantly lower expression levels compared to the WT (*p* < 0.0001 or *p* < 0.001). No significant expression was seen in the control vesicles for either Sf9 or HEK293 (*p* < 0.0001). The size of the detected protein in Sf9-ABCG2 preparations was approximately 70 kDa and slightly higher in the HEK293-ABCG2 preparations possibly due to glycosylation differences the mammalian and insect cells. Two distinct bands could be seen in the Sf9-ABCG2 preparations of S441N and M515R, whereas two bands could be seen in most of the HEK293-ABCG2 vesicles.

**Fig. 5:**
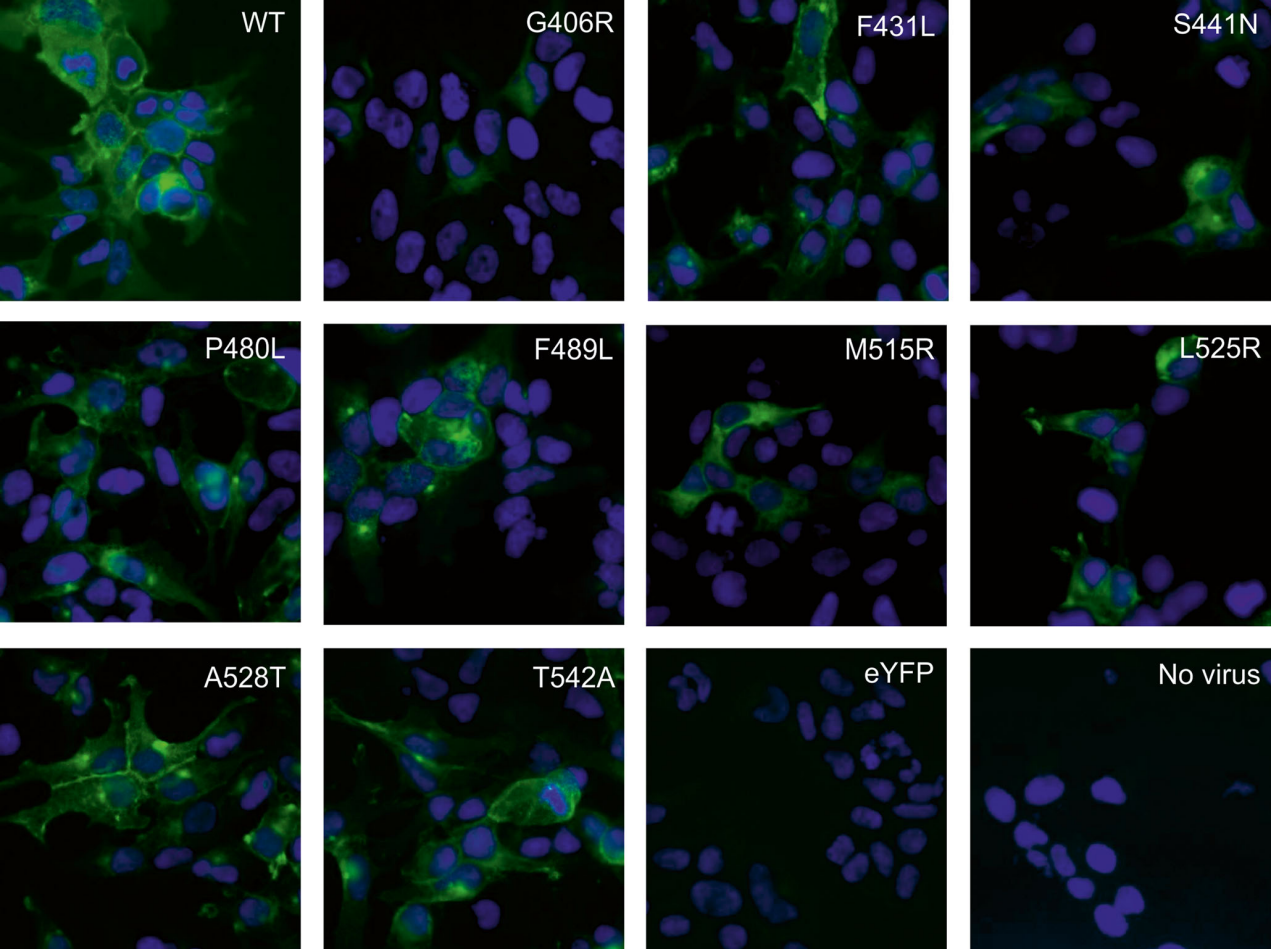
Localization of ABCG2 variants in HEK293 cells transduced with recombinant baculovirus. Immunofluorescence microscopy was used to study the localization of ABCG2 variants as described in the Materials and Methods section. ABCG2 was detected using the BXP-21 antibody and AlexaFluor 488 (*green*) and nuclei were stained with DAPI (*blue*).

Immunofluorescence microscopy was used to study the localization of ABCG2 variants in HEK293 cells. Wild-type ABCG2 transduced in HEK293 cells showed typical membrane staining when probed with the mAB BXP-21 (Fig. 5). All samples transduced with ABCG2 variants showed some ABCG2 expression in line with the results from western blotting. Due to the nature of the baculovirus system, leading to overexpression in affected cells, some ABCG2 was also retained inside the cell. On the other hand, not all cells were transduced by with the virus and expressed ABCG2. No expression of ABCG2 was detected in the control samples of non-transduced cells or eYFP-transduced cells. Cell surface expression, seen as an intensified band-like signal at cell edges, could be observed for variants F431L, P480L, F489L, A528T and T542A. On the contrary G406R, S441N, M515R and L525R did not show an increased signal at the cell surface, but instead tended to accumulate inside the cells or show low expression in general. This corresponds to the low expression seen in the western blot of HEK293-ABCG2 membrane vesicles of variants G406R, S441N and M515R.

## Discussion

In this study, nine naturally occurring variants predicted to be localized in the transmembrane helices of ABCG2 were expressed in both Sf9 insect cells and human derived HEK293 cells. We used these two cell systems because despite insect cells being widely used to study ABC transporters, Sf9-ABCG2 preparations have decreased activity compared to mammalian systems due to the lower cholesterol content in Sf9 cell membranes (26). ABCG2 has been shown to localize in cholesterol-enriched lipid rafts in cell membranes and exhibit reduced transport activity with cholesterol depletion (27). Moreover, insect cells are grown at 27°C and mammalian cells at 37°C. It has been shown in several studies that expression at low temperatures may rescue certain protein variants that were not expressed at 37°C (28). In addition to this, insect cells differ from mammalian cells with regards to post-translational modifications such as glycosylation, which can be detected in western blotting, but glycosylation differences do not appear to affect ABCG2 ATPase activity (26). The activity of the ABCG2 variants studied here relative to the WT ABCG2 were very similar between the Sf9 and HEK293 preparations, despite differences in the relative expression levels. Strikingly, we detected significantly decreased transport of the two test substrates used in this study for all nine variants. Five of these variants have previously been studied *in vitro*, while to our knowledge the activity of the variants G406R, P480L, M515R and T542A has not previously been characterized.

The F431L, S441N and F489L variants are the most studied of the naturally occurring transmembrane SNPs. The F431L and F489L variants have been reported to have substrate dependent changes in transport activity; methotrexate transport was not observed (29), while cells carrying the variants still displayed resistance towards other substrates like mitoxantrone and SN-38, even though the resistance was reduced compared to the WT ABCG2 (15,29,30). In contrast, the S441N variant appears to be unable to transport any of the tested substrates (9,12,15,16,29). These findings correlate well with our results, as we observed some transport activity by the F431L and F489L variants, while no transport activity could be detected by the S441N variant. Moreover, the F431L and F489L variants were expressed on the cell surface in HEK293 cells as has been reported (15,30,31), while the S441N variant was mainly found intracellularly as expected from previous research (12,15,16,32). Based on previous studies in Flp-In-293 cells, the S441N variant appears to be unstable, since the presence of proteasomal proteolysis inhibitors was able to rescue the protein and increase cell surface expression (32). The glycosylation pattern of S441N has been reported to differ from WT ABCG2 and indeed two bands were also seen for S441N in this study, with the main band in the Sf9 preparation being the lower molecular weight form.

The L525R and A528T variants have not been as well characterized. In a study by Skoglund *et al.* (17) both variants were found at the plasma membrane, but the L525R variant had significantly decreased expression and provided low resistance towards tyrosine kinase inhibitors in a cellular assay, while the A528T variant had similar expression levels compared to the WT, but still displayed a decreased resistance towards the tyrosine kinase inhibitors. We attained very similar results, as L525R was unable to transport either of the tested substrates, while A528T was the most active of the tested variants, with a transport activity of approximately half of the WT ABCG2. L525R was mainly retained inside the cells and expression in the HEK293 membrane vesicles was reduced by 75% compared to WT. Immunostaining showed that A528T was correctly localized at the cell membrane, but the expression in vesicles was roughly half of the WT. The relatively high activity of the A528T variant in comparison to the other transmembrane variants might be explained by the localization of the SNP at the edge of the predicted fourth transmembrane helix of ABCG2. If the topological prediction is correct and the amino acid is not positioned within the membrane, this substitution could be well tolerated since both alanine and threonine are fairly small, neutral amino acids at physiological pH.

*In vitro* functionality or expression of the variants G406R, P480L, M515R and T542A has not been reported previously. The transport activity of these variants was significantly impaired in both Sf9 and HEK293 vesicles for LY as well as E_1_S in the HEK293 vesicles. Low activity in membrane vesicles could be partly explained for G406R and M515R by the retention of ABCG2 inside the HEK293 cells seen in the immunofluorescence studies and the western blot. However, it cannot be solely explained by this since transport was low also in Sf9 vesicles despite a 39- and 45-fold higher relative expression level in Sf9 than HEK293 vesicles for G406R and M515R, respectively. G406 is the first of the glycines in the dimerization motif G/A/SXXXG, which is conserved in the ABCG-family (33). Mutation of either of the glycines to leucine impaired substrate transport of ABCG2, while substituting both glycines with alanine did not interfere with the function (33). As the arginine is likely to disrupt the molecular architecture in the transmembrane domain of the protein to an even higher degree than the leucine substitution, it was not unexpected that the G406R variant showed extremely low transport activity, similarly to the M515R and the L525R variants. P480L and T542A did localize at the cell membrane in HEK293 cells, although the expression was lower than WT. The low activity of these variants in the Sf9 vesicles, despite high expression, points to functional defects of the protein. Interestingly, the T542A substitution may display substrate dependent effects on transport activity as it appears able to transport E_1_S but not LY.

The online tools SIFT and Poly-Phen were used to predict the consequences of amino acid substitutions on protein function (Table I) (21,22). Similar effects were predicted for seven of the variants with both services while contrasting predictions are given for the F431L and A528T substitutions. Both programs predicted that four of the nine transmembrane SNPs are tolerated or benign. Since all of the studied variants decreased the apparent function of ABCG2 *in vitro*, it can be concluded that these tools are not capable of predicting the effect of all polymorphisms. Both programs correctly predicted the disruptive effect of the substitutions to arginine, as well as the proline to leucine substitution. However, the programs failed to predict the damaging effect of substitutions where the amino acid properties did not change as much, *i.e.* the phenylalanine –leucine or threonine-alanine and even the serine-aspargine substitution. The poor predictions might be due to the localization of the SNPs in the transmembrane region, which is not considered by the programs. The substitutions are likely to cause improper folding of the protein due to the membrane localization, which is less tolerant to changes in the peptide sequence. Subsequently the misfolded protein is not translocated to the plasma membrane, but remains intracellular.

The global allele frequency of the studied variants is generally low (< 1%) based on genotyping studies in the Japanese population (30,34–36) and the 1000 Genomes Project database (1000genomes.org). This is the case for most ABCG2 SNPs, with the notable exceptions of the Q141K and V12M variants. Due to the low frequency, the effect of the studied variants is unlikely to be identified in genome-wide association studies (GWAS) or clinical studies that are mainly suited to recognize commonly occurring SNPs. However, the observed significant decrease in the *in vitro* transport activity suggests that patients harboring these variant alleles are at risk for adverse effects of ABCG2 substrates or diseases linked to ABCG2 function, such as gout. Since amino acid substitutions in the transmembrane regions do not appear to be well tolerated, it is likely that other nonsynonymous SNPs localized to the membrane helices have similar damaging effects on ABCG2 function.

## Conclusion

In conclusion, we have shown that nine naturally occurring polymorphic variants of ABCG2 display significantly decreased transport function in the vesicular transport assay. The damaging effect of variants G406R, P480L, M515R and T542A is, to our knowledge, reported here for the first time. The findings were confirmed by using two independent expression systems and they are in line with previous literature. Non-functionality could be explained for some variants by altered expression patterns as shown by immunofluorescence microscopy. These results indicate that patients harboring these ABCG2 variant forms could suffer from unexpected pharmacokinetic events of ABCG2 substrate drugs or have an increased risk for diseases where ABCG2 is implicated. This type of *in vitro* characterization of variants is important especially for variants with low allele frequencies which are difficult to pick up in the limited sample populations used in pharmacogenetic studies.

## Electronic supplementary material

Below is the link to the electronic supplementary material.

ESM 1 (DOCX 15 kb)

## ABBREVIATIONS

ABC: ATP-binding cassette
BCRP: Breast cancer resistance protein
E_1_S: Estrone sulfate
HEK293: Human embryonic kidney cell line
LY: Lucifer yellow
Sf9: *Spodoptera frugiperda* derived insect cell line
SNP: Single-nucleotide polymorphism
WT: Wild-type
