# Supplementary material for: Transmembrane Domain Single-Nucleotide Polymorphisms Impair Expression and Transport Activity of ABC Transporter ABCG2
Source: Pharm Res. 2017 Mar 9;34(8):1626–36. doi: 10.1007/s11095-017-2127-1 (PMC5498656; doi:10.1007/s11095-017-2127-1)
Supplement: Supplementary file 1 — (DOCX 15 kb) [file 11095_2017_2127_MOESM1_ESM.docx]

**Supplementary Table I** Primers used for incorporating single nucleotide polymorphisms (SNPs) into the *ABCG2* gene.

| SNP | Amino acid change | Forward primer ^a^ | Reverse primer |
| --- | --- | --- | --- |
| 1216 G>A | G406R | AGTCGTACTG**A**GACTGGTTATAG | GTGACAATGATCTGAGCTATAG |
| 1291 T>C | F431L | TGGGGTTCTC**C**TCTTCCTGAC | GCTCTGTTCTGGATTCCAG |
| 1322 G>A | S441N | TGTTTCAGCA**A**TGTTTCAGCC | CTGGTTGGTCGTCAGGAAG |
| 1439 C>T | P480L | GATTTATTAC**T**CATGAGGATGTTAC | AGATAACAGTTTTCCAAGGAAATAAG |
| 1465 T>C | F489L | AAGTATTATA**C**TTACCTGTATAGTGTAC | GGTAACATCCTCATGGGTAATAAATC |
| 1544 T>G | M515R | ACCCTTATGA**G**GGTGGCTTATTC | AAACATCATAACGAAGAAGGC |
| 1574 T>G | L525R | TCCATGGCAC**G**GGCCATAGCA | ACTGGCTGAATAAGCCACC |
| 1582 G>A | A528T | GGCACTGGCCATA**A**CAGCAGGTCAGAG | CTCTGACCTGCTG**T**TATGGCCAGTGCC |
| 1624 A>G | T542A | ACTTCTCATG**G**CCATCTGTTTTG | GTTGCTACAGAAACCACAC |

^a^ The altered nucleotides are marked in bold.
